# microRNA-25 as a novel modulator of circadian *Period2* gene oscillation

**DOI:** 10.1038/s12276-020-00496-5

**Published:** 2020-09-23

**Authors:** Inah Park, Doyeon Kim, Jeongah Kim, Sangwon Jang, Mijung Choi, Han Kyoung Choe, Youngshik Choe, Kyungjin Kim

**Affiliations:** 1 Department of Brain and Cognitive Sciences, Daegu Gyeongbuk Institute of Science and Technology (DGIST), Daegu, 42988 Korea; 2 Department of Anatomy, Korea University College of Medicine, Seoul, 02841 Korea; 3 Aging Neuroscience Research Group, Korea Brain Research Institute (KBRI), Daegu, 41068 Korea

**Keywords:** Circadian rhythms, RNAi

## Abstract

Circadian clock controls an organism’s biological rhythm and regulates its physiological processes in response to external time cues. Most living organisms have their own time-keeping mechanism that is maintained by transcriptional–translational autoregulatory feedback loops involving several core clock genes, such as *Period*. Recent studies have found the relevance between the modulation of circadian oscillation and posttranscriptional modifications by microRNAs (miRNAs). However, there are limited studies on candidate miRNAs that regulate circadian oscillation. Here, we characterize the functions of novel miRNA-25 regulating circadian *Period2* (*Per2*) expression. Using several in silico algorithms, we identified novel miR-25-3p that, together with miR-24-3p, targets the *Per2* gene. Luciferase reporter assays validated that miR-25-3p and miR-24-3p repressed *Per2* expression and confirmed their predicted binding sites in the 3′-untranslated region (UTR) of *Per2* mRNA. Real-time bioluminescence analyses using Per2::Luc mouse embryonic fibroblasts confirmed that PER2 protein oscillation patterns were responsive to miR-25-3p and miR-24-3. The overexpression of miR-25-3p or miR-24-3p resulted in the dampening and period shortening of the PER2::LUC oscillation, while inhibition of either miRNA increased the relative amplitude of the PER2::LUC oscillation. Notably, endogenous miR-25-3p expression in the suprachiasmatic nucleus (SCN) showed no circadian rhythmicity, but the expression levels differed in various brain regions and peripheral tissues. These results suggest that the posttranscriptional regulation of miR-25-3p and miR-24-3p may differ according to *Per2* gene expression in different tissue regions. In summary, we found that novel miR-25-3p was involved in fine-tuning circadian rhythmicity by regulating *Per2* oscillation at the posttranscriptional level and that it functioned synergistically with miR-24-3p to affect *Per2* oscillation.

## Circadian rhythm: microRNAs keep the clock in order

A newly identified microRNA plays a key role in fine-tuning the genetic interactions governing the circadian rhythms in mammals, according to researchers in South Korea. Numerous studies have suggested that the *Period* genes, which negatively regulate the *CLOCK* and *BMAL1* genes to produce a 24-hour feedback loop, may be further modified by microRNAs after they are transcribed. Kyungjin Kim at Daegu Gyeongbuk Institute of Science and Technology, South Korea, and co-workers confirmed that a novel microRNA, miR-25-3p, reduces the expression of a *Period* gene, *Per2*, in mice. When miR-25-3p is over-expressed, it dampens and shortens the oscillations of *Per2* levels. Interestingly, the researchers showed that natural miR-25-3p expression levels varied across different parts of the brain, supporting the theory that different tissues of the body maintain their own unique circadian cycles.

## Introduction

The circadian rhythm is one of the highly conserved features of the evolutionary process. Each species has its own biological rhythm governed by a master clock, the suprachiasmatic nucleus (SCN) located in the hypothalamus in mammals or an equivalent structure that is reset in an approximate 24-h period according to the rotation of the Earth. This internal biological clock is essential for the synchronization of bodily functions within an organism to maintain homeostasis, such as the sleep/wake cycle, metabolism, thermoregulation, and hormonal regulation^1^. Molecular clockwork involves genetically encoded autoregulatory feedback loops that maintain an approximately 24-h period of circadian oscillation. This self-sustainable biological clock machinery consists of positive and negative transcriptional–translational feedback loops^2^. The core loop of this molecular clock is driven by a heterodimeric transcriptional activator composed of two major clock genes: circadian locomotor output cycle kaput (CLOCK) and brain–muscle–arnt-like protein 1 (BMAL1)^3^. These heterodimers accelerate E-box-mediated transcription and increase the gene expression of negative regulators [such as *Periods* (PERs: PER1, PER2, and PER3) and *Cryptochromes* (CRYs: CRY1 and CRY2)] and circadian output genes. Expressed and dimerized PER:CRY represses the transcriptional activity of CLOCK:BMAL1, thereby downregulating their own gene transcription. As a result, these molecular feedback loops generate a 24-h circadian oscillation within an organism^4,5^.

In addition to the basic transcriptional–translational feedback loops, the oscillation of the molecular circadian clock is finely regulated by posttranscriptional and posttranslational modifications. In the present study, we investigated the posttranscriptional regulation of the *Per2* gene, a negative regulator in the molecular clock machinery. Some small noncoding mature miRNAs of generally 21–25 nucleotides have been associated with the fine-tuning mechanisms of circadian oscillation^6–8^. The maturation of functional miRNAs is completed by endoribonuclease DICER in the cytoplasm, and each mature miRNA contains a seed sequence of 7 or 8 nucleotides that binds to its complementary region(s) on target mRNAs. A functional miRNA can interact with hundreds of target mRNAs to exert various levels of regulatory effect^9^, most by transcriptional repression in mammals^10^. A mature miRNAs bind to the 5′- or 3′-untranslated region (UTR) of target mRNAs with the assistance of Argonaute (AGO) proteins and cause translational repression by preventing the initiation of translation^11,12^. Chen et al.^7^ demonstrated the involvement of miRNAs in the expression of circadian molecular clock genes using DICER-deficient cells and conditional DICER-knockout mice. Their results revealed that the absence of miRNAs did not disrupt the generation of circadian rhythmicity; in contrast, it resulted in a significant shortening of the circadian period length^13–15^. Moreover, Chen et al.^7^ found that *Per2* was the only affected core clock gene, with a slight elevation of its mRNA and a significant increase in PER2 protein levels. These findings support the previous understanding suggesting a role for the *Per2* gene in circadian rhythm generation; i.e., PER2 is a negative regulator critical for modulating circadian rhythmicity in the approximate 24-h period^16^.

However, the mechanism underlying the fine-tuning of circadian molecular oscillations via posttranscriptional modification by multiple miRNAs remains unknown. Here, we aimed to identify a miRNA that directly affects the expression of PER2 and determine the roles of multiple miRNAs in fine-tuning the molecular clockwork by comparing their impacts on temporal patterns of clock gene expression.

## Materials and methods

### Animals

For circadian time point samples, 8-week-old C57BL/6J male wild-type (WT) mice were obtained, and Period2::Luc knock-in (Per2::Luc KI) mice were sacrificed on postnatal days 7–9 to obtain samples for a brain slice culture. The mice were housed under a 12-h/12-h light-dark photoperiod at a constant temperature (22–23 °C). All procedures were conducted in compliance with the rules and regulations established by the Institutional Animal Care and Use Committee (IACUC) of Daegu Gyeongbuk Institute of Science and Technology (DGIST).

### Cell culture and transfection

Per2::Luc knock in mouse embryonic fibroblasts (Per2::Luc KI MEFs), wild-type MEFs, wild-type NIH3T3 and *Per2*-promoter-driven Luc mutant NIH3T3 cells were maintained in Dulbecco’s modified Eagle’s medium (DMEM, Gibco, USA) supplemented with 10% fetal bovine serum (Gibco) and 1% antibiotic–antimycotic (AA, Gibco). The cells were cultured in a humidified incubator that was maintained at 37 °C in a 5% CO_2_ environment. The Per2::Luc KI MEFs were prepared from Per2::Luc KI transgenic mice carrying a luciferase reporter protein that was fused in-frame within an endogenous PER2 protein at the C-terminus^17^. Reporter gene expression enabled real-time monitoring of PER2 protein dynamics with the intact 3′-UTR with *Per2* miRNA-binding sites. Prior to transfection, cells were seeded in plates or dishes and allowed to grow for 24 h. To overexpress or inhibit selected miRNAs, AccuTarget miRNA mimics and inhibitors (Bioneer, South Korea) were prepared and transfected using Lipofectamine 3000 transfection reagent (Invitrogen, USA) according to the manufacturer’s instructions. Synthetic inhibitors for miR-24-3p and miR-25-3p were produced based on the miRbase sequence database (http://www.mirbase.org); the accession numbers were MIMAT0000219 and MIMAT0000652, respectively. For the control groups, a miR-control oligomer (Bioneer) was used. To examine the effects of these miRNAs on Per2 transcription, Per2::Luc KI MEFs were transfected with miR-control-, miR-24-3p-, or miR-25-3p-OE vectors (Applied Biological Materials, Canada) followed by dexamethasone (DEX) synchronization. Thirty hours after DEX synchronization, the cells were incubated with actinomycin D (at a final concentration of 1 µg/ml) or DMSO and then harvested at 0 or 6 h after drug treatment for use in the downstream measurements.

### Organotypic slice culture

An organotypic slice culture was prepared as described previously^18^, with some modifications. Briefly, the brains of neonatal mice (postnatal days 7–9) were obtained and rapidly placed in ice-cold Gey’s balanced salt solution supplemented with 10 mM HEPES and 30 mM glucose, bubbled with 5% CO_2_ and 95% O_2_. Coronal slices (400 μm thickness) were prepared using a vibratome (Leica, Nussloch, Germany). The slices were placed onto a culture membrane (Merck Millipore, USA) with 50% minimum essential medium, 25% Gey’s balanced salt solution, 25% horse serum, 36 mM glucose, and 100 U/mL antibiotic–antimycotic. The medium was replaced every 3 days before the experiments were performed. Then, the slice cultures were premeasured for PER2::LUC expression in recording medium supplemented with d-luciferin (Promega, USA) at a final concentration of 100 µM using a real-time bioluminescence recording device (Kronos Dio, Japan). Each SCN slice culture was transduced with in-house generated lentiviral vector OE miR-control-GFP, miR-24-3p-GFP, and/or miR-25-3p-GFP and incubated for 1 week. Then, the lentiviral transduction effect was recorded using the real-time bioluminescence device.

### DNA constructs and plasmids

The full-length and truncated 3′-UTRs of *Per2* were amplified from genomic DNA using the primers indicated. Each fragment was cloned into a plasmid with a pGL3 promoter (Promega). The following primers were used: *Per2* 3′-UTR forward for both fragments, 5′-AGA TCG CCG TGT AAT TCT AGA ATT AGA CGG TGC TCG GAA GA-3′; *Per2* 3′-UTR truncated reverse, 5′-TCT CAA GGG CAT CGG TCG ACT CCT CAC TGG TGA TGT CTC G-3′; and *Per2* 3′-UTR full-length reverse, 5′-TCT CAA GGG CAT CGG TCG ACG ACA CAA GCA GTC ACA CAA-3′. The pGL3 promoter plasmid was 5 kb, and the inserted 3′-UTRs were confirmed by gel electrophoresis based on their respective size of 6.8 kb (*Per2* 3′-UTR full-length) and 6 kb (*Per2* 3′-UTR truncated). The following mutations were introduced into the 3′-UTR of *Per2* for the site-directed mutagenesis study: miR-24-3p, CUGAGCC → CCGCGCC^7^; miR-25-3p, GTGCAAU → GCGCGGU. The inserted fragments were sequenced (Cosmo Genetech, Korea) and validated using the NCBI BLASTN program (Fig. S3). miR-24-3p- and miR-25-3p-OE vectors and the control vector were purchased for the production of lentiviral vectors (Applied Biological Materials).

### Luciferase reporter assay

Wild-type NIH3T3 fibroblasts were seeded in 96-well plates at 1 × 10^4^ cell per well and then cotransfected with pRL-TK (50 ng per well), 3′-UTR of *Per2* inserted into a pGL3-promoter vector (150 ng per well) and miR-control oligomer or miR-24-3p and miR-25-3p mimics or inhibitors (50 nM). After 48 h of transfection, luciferase activity levels were measured using a Dual-Glo luciferase assay system (Promega). Renilla luciferase activity was used to normalize the firefly luciferase activity.

### Real-time bioluminescence monitoring

Per2::Luc KI MEFs and mutant NIH3T3 fibroblasts were plated in 35-mm dishes (Falcon, United States) at a 1.5 × 10^5^ cell density. After 24 h of incubation, the cells were transfected with selected miRNAs (50 nM) using Lipofectamine 3000 according to the manufacturer’s protocol and incubated for 24 h (Invitrogen). The cells were synchronized by 200 nM DEX treatment for 2 h, and the bioluminescence was recorded for 5 days with a real-time bioluminescence device (Kronos Dio) in recording medium with 100 µM d-luciferin (Promega). Light emissions were integrated for 1 min at intervals of 10 min using a dish-type wheeled luminometer. The raw data presented were normalized based on the first minimum bioluminescence point and detrended bioluminescence time series data were obtained by subtracting the 12-h moving average from the luminescence intensity with the background eliminated.

### RNA isolation and real-time quantitative PCR

Total RNA, including small RNAs, was isolated using the Qiagen miRNeasy mini kit (Qiagen, Germany) according to the manufacturer’s protocol. The isolated total RNA was reverse transcribed using an miScript II RT Kit (Qiagen) to quantify both the mRNAs and miRNAs in the total RNA sample. The miRNA expression profiles were analyzed by quantitative polymerase chain reaction (qPCR) using the miScript SYBR Green PCR kit (Qiagen) with designed primers (Qiagen), Mm_miR-24_1 miScript primer assay, Mm_miR-25_1 miScript primer assay and Hs_SNORD95_11 miScript primer assay for miR-24-3p, miR-25-3p and the internal control SnoRD95, respectively. The following primer sequences were used for mRNA quantification: *Per2* up, 5′-ATCCCACGAACACCTCATGA-3′; *Per2* dn, 5′-CCCTGAGCTGTCCCTTTCTA-3′; *Tbp* up, 5′-GGG AGA ATC ATG GAC CAG AA-3′; *Tbp* dn, 5′-CCG TAA GGC ATC ATT GGA CT-3′.

### Western blotting

Cells and tissue samples were washed with prechilled phosphate-buffered saline (PBS) and lysed in radioimmunoprecipitation assay buffer [150 mM NaCl, 0.1% Triton X-100, 0.5% sodium deoxycholate, 0.1% sodium dodecyl sulfate, and 50 mM Tris-HCl (pH 8.0)] with Halt protease and phosphatase inhibitor cocktail (100×) (Thermo Scientific, USA) and sonicated in an ice bath. Supernatants were obtained, and total proteins were quantified using a Pierce BCA protein assay kit (Thermo Scientific). For each sample, 10 µg of protein was loaded and separated by sodium dodecyl sulphate-polyacrylamide gel electrophoresis (7.5% gel) and transferred to a polyvinylidene fluoride membrane (Merck Millipore). Immunoblot analyses were carried out with rabbit anti-PER2 polyclonal antibody (Santa Cruz Biotechnology, USA) and anti-beta-ACTIN-HRP (Santa Cruz Biotechnology).

### Immunohistochemistry and confocal imaging

Transduced SCN slice cultures were fixated with a prechilled 4% paraformaldehyde (PFA) solution and washed with PBS. For immunostaining, tissue samples were incubated with a DeepLabel antibody staining kit (Logos Biosystems, South Korea) permeabilization solution for 6 h and then labeled overnight with primary antibodies: rabbit-anti-PER2 (1:500, Merck Millipore), mouse-anti-NeuN (1:500, Merck Millipore), and DAPI (1:10,000, Thermo Scientific) at 37 °C with agitation. After washing with PBS three times, the appropriate secondary antibodies, donkey anti-rabbit-Alexa-594 and donkey anti-mouse-Alexa-647 (Invitrogen), were applied for 6 h at room temperature. The slice tissues were then imaged using a confocal LSM700 microscope (Carl Zeiss, Germany).

### Statistical analysis

Statistical analyses were performed using Prism 8 (GraphPad Software, USA). For the luciferase reporter assay and real-time bioluminescence analyses, Cosinor software (https://www.circadian.org/softwar.html) was used for analyzing each period of detrended data format. To calculate the area under the curve, the data between the first and second nadirs from the raw data were analyzed using Prism 8 (Fig. S1). Student’s *t* test or one-way analysis of variance (ANOVA) was performed to assess the significance of intergroup differences. Significance was attributed for *p* values < 0.05. The results are represented as the means ± standard error (SE). In general, the bioassays were run in duplicate and repeated (noted as “*n*” in figure legends) for different occasions. The details of each statistical test used (statistics, significance levels, sample sizes and SEs) are described in each figure legend.

## Results

### Identification of miR-25-3p, a novel microRNA targeting the 3′-UTR of mouse *Per2* mRNA

**Fig. 1:**
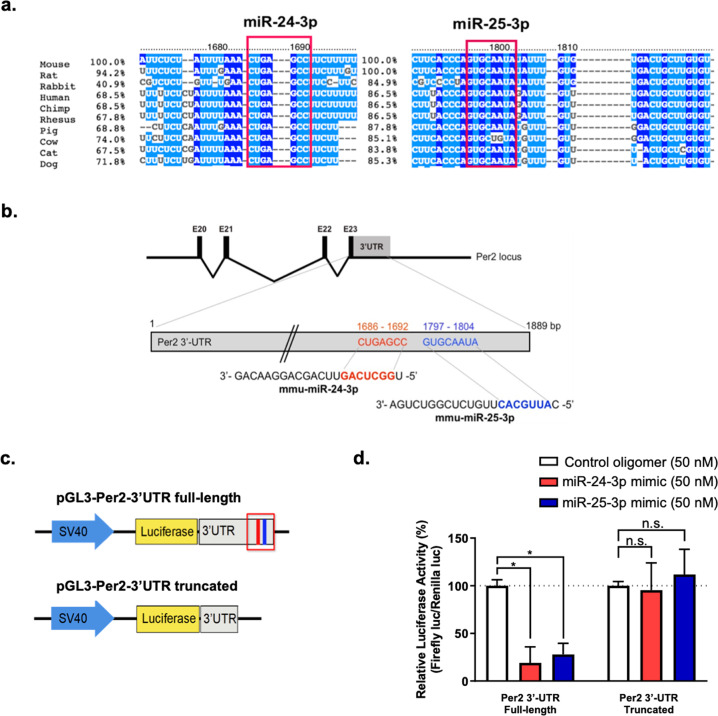
Candidate microRNAs targeting the 3′-untranslated (UTR) region of *Period2 (Per2)*. **a** Conserved miR-24-3p and miR-25-3p binding sites on the 3′-UTR of *Per2* in several vertebrates (indicated with red outlines). **b** Predicted binding sites of miR-24-3p (red) and miR-25-3p (blue) are illustrated on the 3′-UTR of *Per2*, and the 3′-UTR targeting sequences of miR-24-3p and miR-25-3p are indicated. **c** Schematic of the constructed pGL3 vectors with binding sites on the 3′-UTR of *Per2* for the full-length or truncated miR-24-3p and miR-25-3p. Predicted binding sites of miR-24-3p (red bar) and miR-25-3p (blue bar) are illustrated on the 3′-UTR of *Per2*. **d** NIH3T3 fibroblasts were cotransfected with pRL-TK and either a constructed pGL3 vector carrying a miR control oligomer (50 nM), miR-24-3p mimic (50 nM, red), or miR-25-3p mimic (50 nM, blue). Data are presented as the means ± SE (*n* = 4), and significance was assessed by Student’s *t* test (*p < 0.01).

Three in silico databases, namely, TargetScan, miRDB, and DIANA microT-CDS, were screened for miRNAs that target the 3′-UTR of mouse circadian *Per2* mRNA. miR-25-3p and miR-24-3p were selected as references based on their higher binding affinity for the 3′-UTR of *Per2* mRNA (their scores were approximately 90% or greater; data not shown). The complementary sequences of the miR-25-3p and miR-24-3p binding-sites on the 3′-UTR of *Per2* mRNA were highly conserved among several vertebrates (Fig. 1a). After selecting miR-25-3p and miR-24-3p, their predicted putative recognition sites on the 3′-UTR of *Per2* were identified using TargetScan. The complementary sequences of the seed miR-25-3p and miR-24-3p sequences were predicted to be on the 3′-UTR of *Per2* mRNA at 1797–1804 and 1686–1692 nucleotides, respectively (Fig. 1b). Based on these predictions, two luciferase vectors were constructed: full-length 3′-UTR of *Per2* (Per2 3′-UTR full-length) and 3′-UTR with truncated miR-25-3p and miR-24-3p binding sites (*Per2* 3′-UTR truncated), as depicted in Fig. 1c. To determine whether miR-25-3p and miR-24-3p interact with their predicted complementary regions in the 3′-UTR of *Per2*, a dual-luciferase assay was conducted with NIH3T3 fibroblasts, and pRL-TK was as a control for normalization. The relative luciferase activity levels of the *Per2* 3′-UTR full-length constructs were significantly attenuated by the miR-25-3p mimic or miR-24-3p mimic, compared to the effect of the miR-control oligomer treatment, while the expression of the truncated *Per2* 3′-UTR construct was not affected (Fig. 1d). These results confirmed that miR-25-3p and miR-24-3p directly interact with the 3′-UTR of *Per2* and regulate gene expression.

### miR-25-3p and miR-24-3p modulate *Per2* gene expression at the posttranscriptional level

**Fig. 2:**
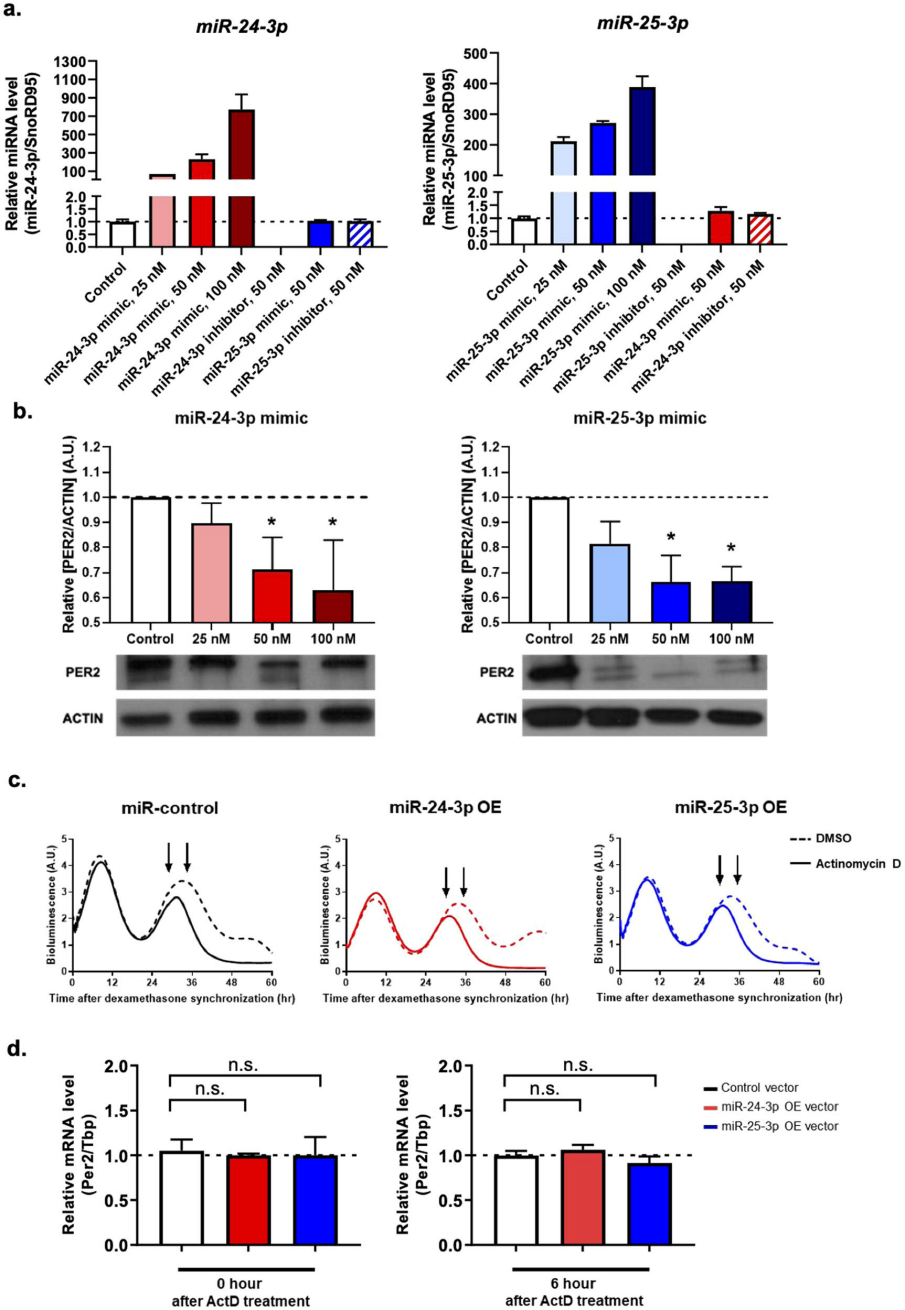
miR-24-3p and miR-25-3p modulate *Per2* gene expression at the posttranscriptional level. Wild-type mouse embryonic fibroblasts (WT MEFs) were transfected with miR-24-3p or miR-25-3p mimics in a dose-dependent manner (25, 50, and 100 nM) and then assayed for the expression levels of **a** miR-24-3p and miR-25-3p using real-time qPCR (*n* = 4) and **b** quantified PER2 protein expression by Western blotting (*n* = 3). To determine the effects of miR-24-3p and miR-25-3p on *Per2* transcription, Period2::Luc knock-in mouse embryonic fibroblasts (Per2::Luc KI MEFs) were transfected with 0.5 µg of miR-24-3p- and miR-25-3p-overexpressing and control vectors, and then, actinomycin D (a potent transcription inhibitor) was added for 30 h after dexamethasone synchronization. **c** The results of real-time bioluminescence recordings are represented in raw data format after actinomycin D (solid line) and DMSO (dashed line) treatment. **d** *Per2* mRNA was quantified after an additional 0 and 6 h of actinomycin D treatment (Per2::Luc KI MEFS were harvested as indicated by the arrowheads), and the data were normalized to that of the control vector (*n* = 4). Data are presented as the means ± SE, and the significance was assessed by one-way ANOVA, *p < 0.05 compared to the control groups.

To measure the changes in miR-25-3p and miR-24-3p expression levels after transfecting each oligomer into WT MEFs at final concentrations of 25, 50, and 100 nM, real-time qPCR was performed (Fig. 2a). The dose-related fold increase over that of the control was exceptional. The large increase in miRNA levels after transfection was consistent with recent literature^19,20^. When transfecting the inhibitors, the target miRNAs were detected to be near zero, indicating the successful inhibition of the miRNAs of interest using their inhibitors. The expression levels of the target miRNAs compared to the that of the control group suggested that both primers, miRNA mimics, and inhibitors were specific to their targets. The optimal concentration of miRNA oligomers required to modulate the expression of PER2 protein was determined by Western blotting (Fig. 2b). Starting from the transfection dose at 50 nM, a decrease in PER2 protein levels was considered statistically significant.

To verify that miR-25-3p and miR-24-3p regulate *Per2* after transcription, Per2::Luc KI MEFs were treated with actinomycin D, a potent transcription inhibitor, after transfecting 0.5 µg of miR-overexpressing (OE) vectors (Fig. 2c). The MEFs were treated with actinomycin D for 30 h after dexamethasone synchronization and harvested at 0 and 6 h of actinomycin D treatment (Per2::Luc KI MEFs were harvested as indicated by the arrowheads in Fig. 2c) and assayed for *Per2* mRNA levels. As expected, no differences in *Per2* mRNA levels were found in the miR-25-3p- and miR-24-3p-OE MEFs before and after actinomycin D treatments compared to level in the control groups (Fig. 2d). Overall, miR-25-3p and miR-24-3p overexpression did not alter *Per2* transcription but modulated the *Per2* gene at the posttranscriptional level.

### miR-25-3p regulates circadian PER2 oscillation

**Fig. 3:**
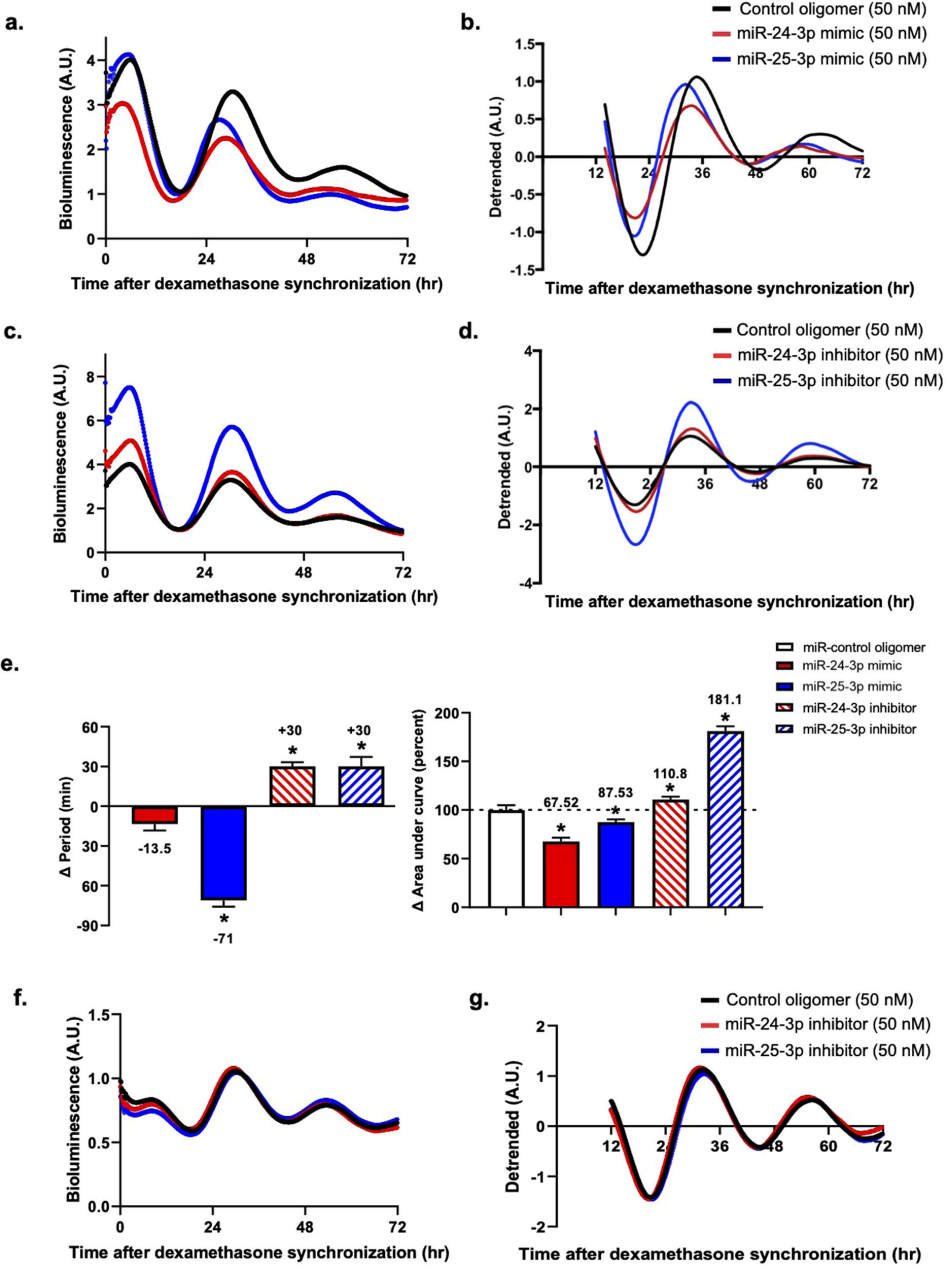
Effects of miR-24-3p or miR-25-3p on the oscillation of the PER2 protein. The results of real-time bioluminescence recordings are represented in raw and detrended data formats. **a**, **b** Per2::Luc KI MEFs were transfected with miR-24-3p or miR-25-3p mimics for gain-of-function studies. **c**, **d** For loss-of-function studies, miR-24-3p or miR-25-3p inhibitors were transfected into the Per2::Luc KI MEFs. Bioluminescence patterns were recorded for 3 days and normalized by the control group. **e** Relative periods and area under curve were calculated from the raw data, which is presented in bar graphs. **f**, **g** *Per2* promoter-driven luciferase (Per2pro-Luc) mutants were transfected into NIH3T3 fibroblasts with an miR-24-3p inhibitor, miR-25-3p inhibitor, or miR-control oligomer. Data are presented as the means ± SE (*n* = 4), and significance was assessed by one-way ANOVA, *p < 0.01 compared to the control group.

To determine the effect of miR-25-3p or miR-24-3p overexpression on rhythmic *Per2* gene expression, Per2::Luc KI MEFs were utilized for real-time bioluminescence analysis. Per2::Luc KI MEFs were constructed with a luciferase reporter inserted in frame at the end of exon 23 of the *Per2* gene, followed by an endogenous 3′-UTR sequence^17^. In summary, the Per2::Luc KI MEFs constituted an appropriate model system to study real-time *Per2* expression as modulated by miR-25-3p or miR-24-3p in vitro. Interestingly, the Per2::Luc KI MEFs transfected with the miR-25-3p or miR-24-3p mimic showed not only shortened periods, by 71 and 13.5 min, respectively, but also a significant dampening of their relative amplitudes, by 87.53% and 67.52%, respectively (Fig. 3e). According to comparison studies of their nadirs and first peaks, the relative differences in phase advancement by both the miR-25-3p and miR-24-3p mimic treatments were increased (Fig. 3a, b). These results demonstrated that the miR-25-3p and miR-24-3p mimic treatments led to the dampening of PER2 expression and phase advancements at different levels. Synthetic miR inhibitors function as antagonists to their endogenous miRNAs by binding to the complementary sequences corresponding to the seed sequences in the target miRNAs^21^. To achieve loss of function, Per2::Luc KI MEFs were transfected with either a miR-25-3p or miR-24-3p inhibitor. As expected, the miR-25-3p and miR-24-3p inhibitors caused a significant delay in the circadian periods, by 30 min in both cases, and a significant increase in the relative amplitudes of PER2 expression, by 181.1% and 110.8%, respectively (Fig. 3c–e). Similar to DICER-KO experiments^7^, the endogenous PER2 protein levels were significantly increased when the 3′-UTR of the *Per2* mRNA-targeting miRNAs was blocked. Mutant Per2::Luc NIH3T3 fibroblasts transfected with miR-25-3p or miR-24-3p inhibitors were used for real-time bioluminescence analyses. In contrast to the Per2::Luc KI MEFs in the previous experiments, the mutant Per2::Luc fibroblast contained no *Per2* 3′-UTR in the luciferase reporter construct, but the expression of the luciferase reporter was driven by the *Per2* promoter. Despite transfecting the miR-25-3p and miR-24-3p inhibitors into mutant Per2::Luc NIH3T3 fibroblasts, no significant changes in the circadian periods or the amplitudes of PER2 expression were observed compared to the control group (Fig. 3f, g). Taken together, the data indicate that *Per2* expression can be altered through its 3′-UTR by either miR-25-3p or miR-24-3p.

### miR-25-3p and miR-24-3p function in a synergistic manner by binding their seed sequences to the complementary target in 3′-UTR of *Per2*

**Fig. 4:**
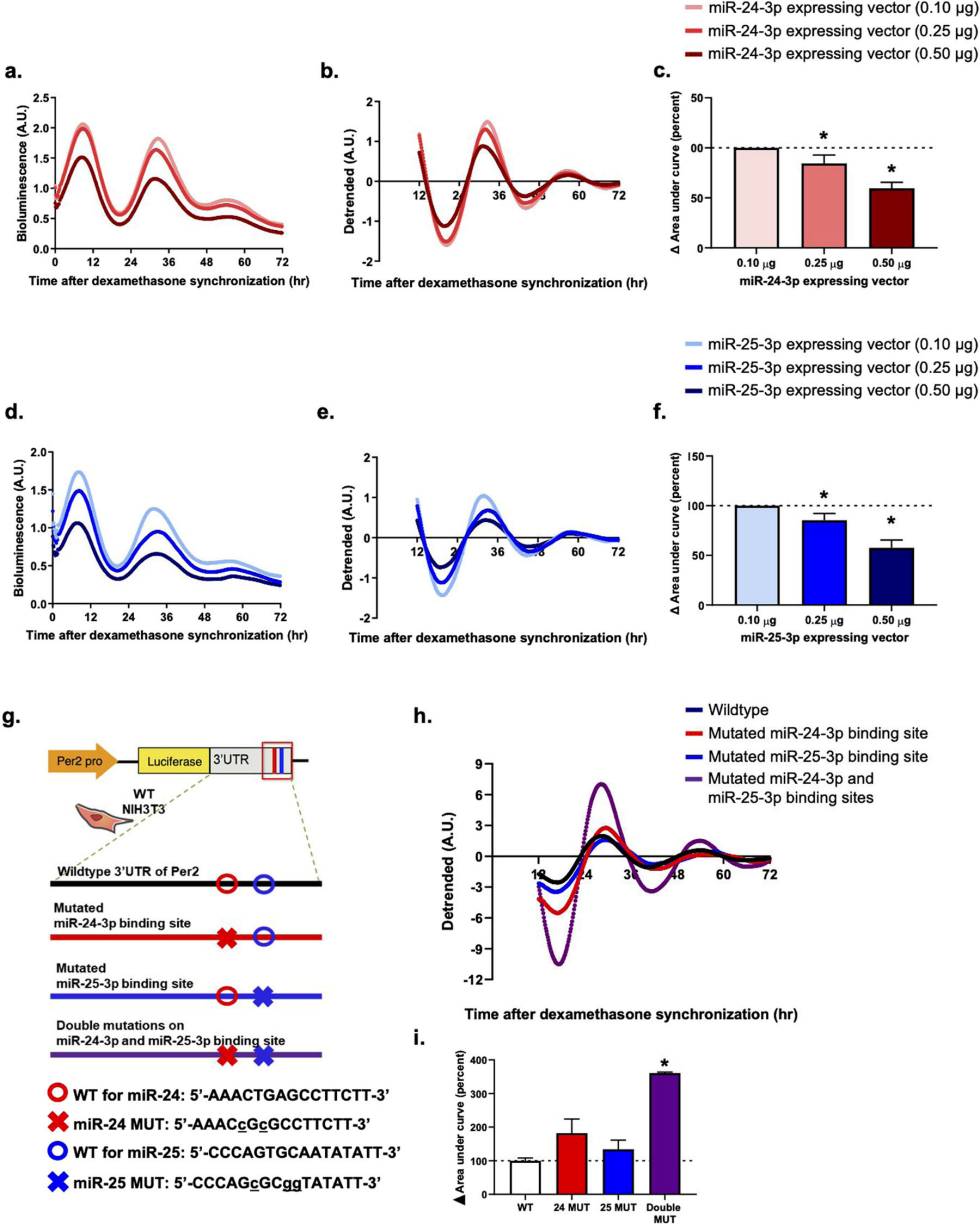
miR-24-3p and miR-25-3p functioned in a dose-dependent manner and bind to their specific sites on the 3′-UTR of *Per2*. Real-time bioluminescence recordings of PER2::LUC oscillation in Per2::Luc KI MEFs that were transfected with **a**, **b** miR-24-3p-overexpressing vectors or **d**, **e** miR-25-3p-overexpressing vectors in a dose-dependent manner. **c**, **f** The area under the curve was calculated from the raw data, which is presented in bar graphs. **g** Schematic for designed site-directed mutations of the miR-24-3p- and/or miR-25-3p binding sites on the 3′-UTR of *Per2* in the pGL3-LUC vector driven by the *Per2* promoter. **h** Results of the real-time bioluminescence recordings of the transfected site-directed mutated luciferase vectors in the NIH3T3 wild-type fibroblasts and **i** the area under curve was calculated, which is presented in bar graphs. Data are presented as the means ± SE (*n* = 3), and significance was assessed by one-way ANOVA, *p < 0.05 compared to the control group.

Herein, we evaluated the effect of the primary forms of miR-25-3p and miR-24-3p using miRNA-expressing vectors in a dose-dependent manner (Fig. 4a–f). Similar to the mature miRNA oligomer treatments, the CMV promoter-driven overexpression of miR-25-3p and miR-24-3p resulted in a slight shortening of the PER2 period and dampening of the PER2 amplitude in a dose-dependent manner. However, the different levels of PER2 oscillation altered by each OE miRNA suggest that there are specific optimal concentrations of miR-25-3p and miR-24-3p for modulating *Per2* expression.

To validate the site-specific effects of miR-25-3p and miR-24-3p on *Per2* expression, we generated luciferase vectors driven by the *Per2* promoter with site-directed mutations in the 3′-UTR of *Per2* corresponding to the seed sequences of miR-25-3p and/or miR-24-3p (Fig. 4g). Compared with that of the control vector and the wild-type 3′-UTR of *Per2*, the period of luciferase expression of the site-mutated 3′-UTR of *Per2* was lengthened. The luciferase vector with double mutations in the miR-25-3p- and miR-24-3p-binding sites showed a greater increase in luciferase amplitude compared to the 3′-UTR of *Per2* vectors with single mutations of either the miR-25-3p- or miR-24-3p-binding site (Fig. 4h, i). This finding suggests that miR-25-3p and miR-24-3p have synergistic effects on *Per2* expression and that each miRNA can function independently of each other.

### Endogenous miR-25-3p and miR-24-3p show no circadian oscillations in the slice cultures and brain tissues

**Fig. 5:**
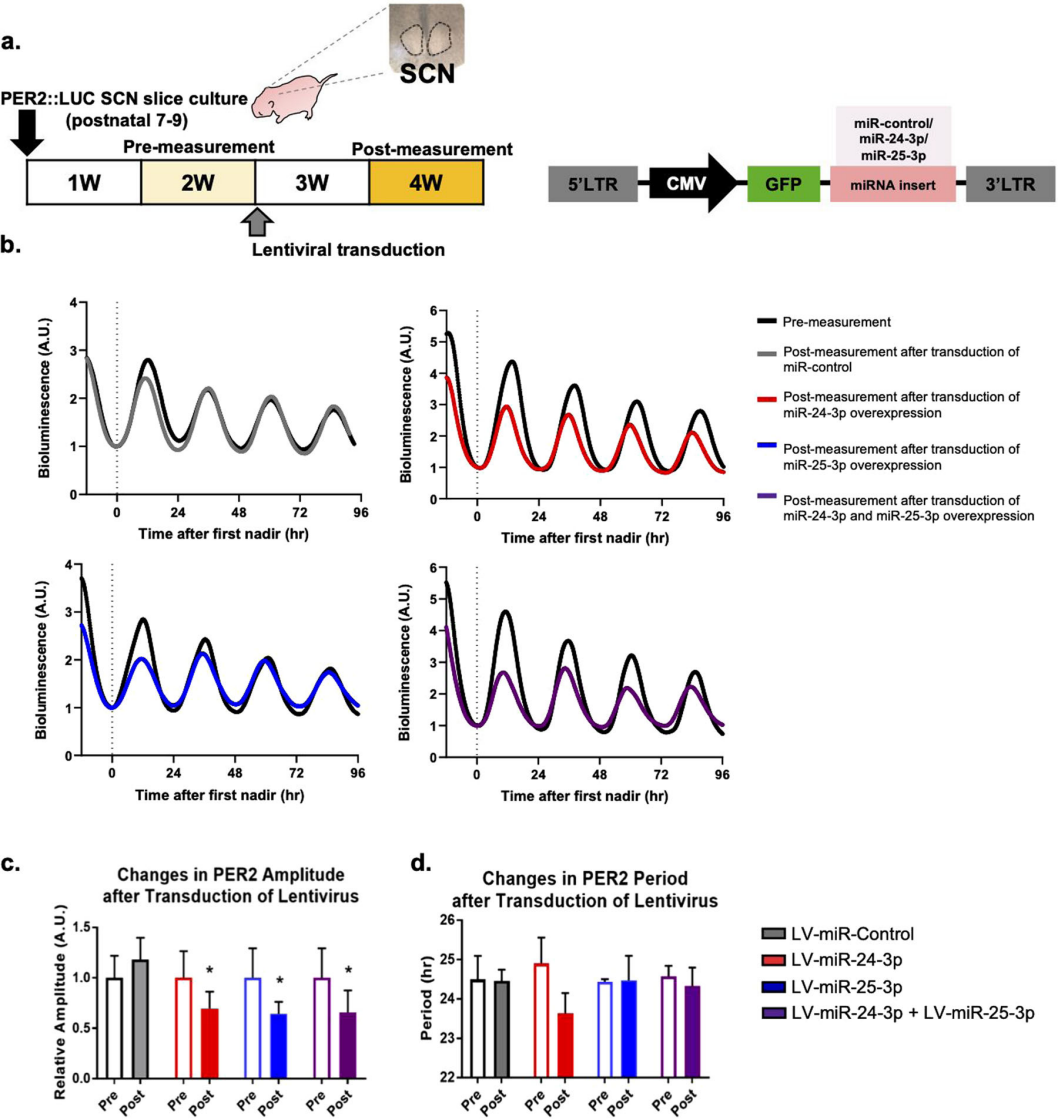
Transduction of lentivirus overexpressing miR-24-3p and/or miR-25-3p dampens the PER2::LUC rhythm in the neonatal SCN slice cultures obtained from the PER2::LUC knock in transgenic mice. **a** Experimental design for transducing CMV-promoter driven lentivirus-miR-control/miR-24-3p/miR-25-3p-GFP in neonatal suprachiasmatic nucleus (SCN) slice cultures. **b** Representative results of PER2::LUC oscillation measured by a real-time bioluminescence recording device. The bioluminescence patterns were aligned to the first nadir of data acquired to compare changes in the expression patterns of PER2::LUC in the pre- and postlentiviral transduced SCN slice cultures (experiments were performed three independent times, *n* = 3). **c** Changes in PER2 amplitudes and periods are presented as the means ± SE. Significance was assessed by Student’s *t* test, *p < 0.01 compared to the prelentiviral transduction conditions.

To explore the effects of miR-25-3p and miR-24-3p on the central master clock ex vivo, we cultured the SCN regions derived from Per2::Luc knock in mice at postnatal days 7–9, and the PER2::LUC oscillation was measured with real-time a bioluminescence recording. The SCN culture samples retained the endogenous neuronal networks within the SCN regions of the hypothalamus^22^. Plasmid vectors OE miR-25-3p or miR-24-3p (Fig. 4a–f) were used to generate the lentiviral vectors transduced into SCN slice cultures during a 1-week incubation period. To minimize experimental errors, the changes in PER2::LUC oscillations were compared for each SCN slice culture by comparing the measurement of the bioluminescence before lentiviral transduction and measurement of the recordings after the 1-week incubation with the miR-25-3p- and miR-24-3p-OE lentiviruses for transduction (Fig. 5a, left). Figure 5b shows the results of the miR-25-3p- and/or miR-24-3p-OE lentiviral of vectors on PER2::LUC oscillations in the SCN slice cultures. Similar to the in vitro results, there were significant changes in the amplitudes of the PER2 levels when miR-25-3p and/or miR-24-3p were overexpressed (Fig. 5c). However, the differences in PER2 periods were not affected, as observed in the in vitro experiments (Fig. 5d).

**Fig. 6:**
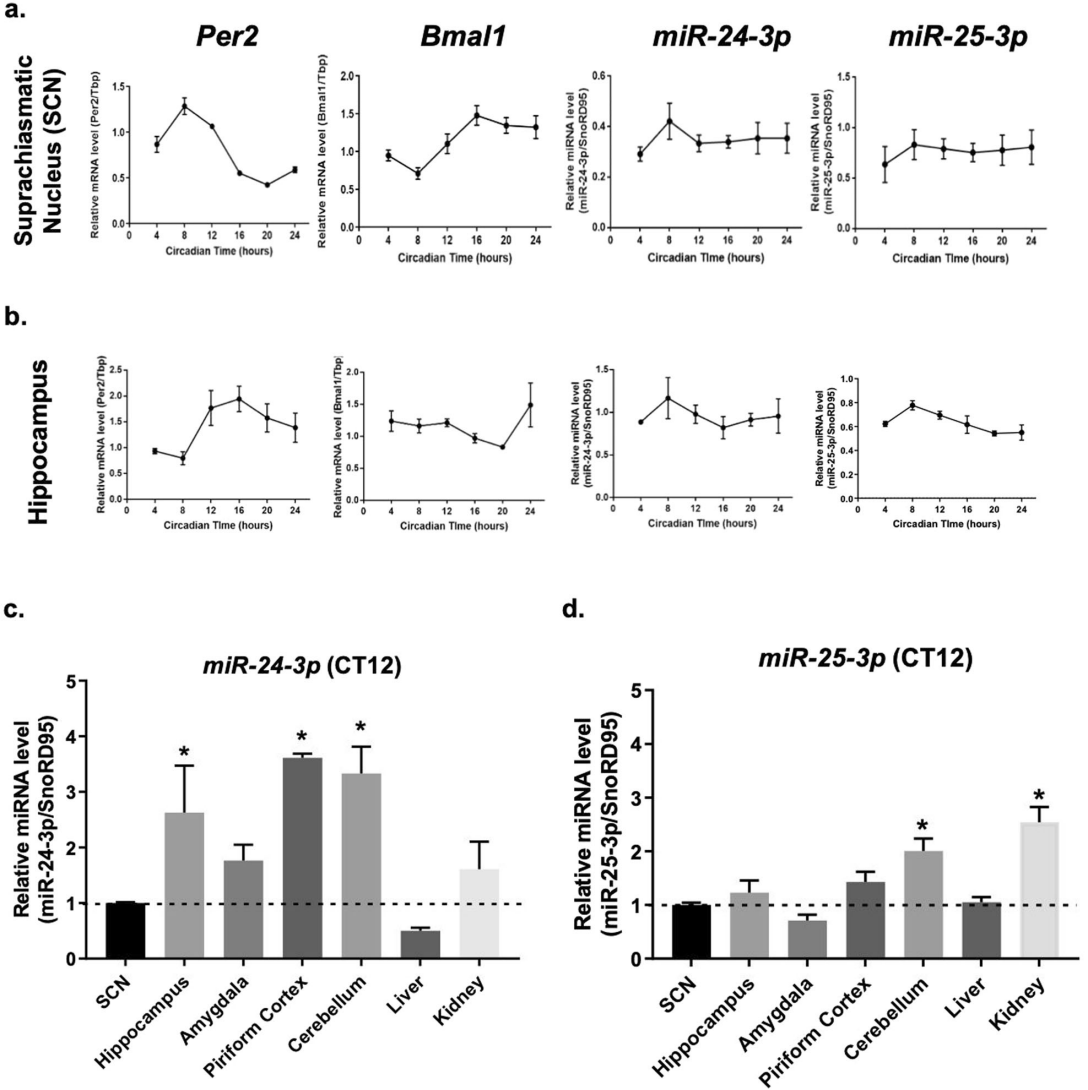
Expression levels of miR-24-3p and miR-25-3p vary in brain regions and peripheral organs. WT mice housed under constant dark conditions for seven days were sacrificed at CT04, 08, 12, 16, 20, and 24 h for measurements of the indicated brain and peripheral tissues. Expression profiles of the circadian clock genes (*Per2* and *Bmal1* mRNAs), miR-24-3p, and miR-25-3p in the **a** SCN and **b** hippocampal brain tissues were obtained by real-time qPCR with the relative quantification method. Expression levels of **c** miR-24-3p and **d** miR-25-3p in various brain and peripheral tissues were examined. CT12 tissue samples were used and normalized to the SCN miR-24-3p or miR-25-3p level. Data for the *Per2* and *Bmal1* mRNAs were normalized by the TATA-box binding protein (*Tbp*) housekeeping gene, while the miR-24-3p and miR-25-3p data were normalized by small nucleolar RNA, C/D Box 95 (*SnoRD95*). Error bars represent the means ± SE of A.U. for each time point measured in three independent measurements. Significance was assessed by one-way ANOVA, *p < 0.05 compared to the control group.

To investigate the endogenous expression patterns of miR-25-3p and miR-24-3p, we quantified the mRNAs of *Per2*, *Bmal1*, miR-25-3p, and miR-24-3p in the SCN, a central clock region, and in the hippocampus, one of the local clock regions in the brain, at various circadian time points (Fig. 6a, b). Regardless of the clock gene mRNA oscillations in each brain region, neither miR-25-3p nor miR-24-3p showed rhythmic expression patterns, as they were expressed constitutively. Considering these nonrhythmic expression patterns of miR-25-3p and miR-24-3p, we further evaluated the expression levels of each miRNA in various brain regions and peripheral tissues at the fixed circadian time point, CT12. The expression levels of miR-24-3p were significantly higher in three regions, namely, the hippocampus, piriform cortex, and cerebellum (Fig. 6c), while miR-25-3p was more highly expressed in the cerebellum and kidney than in the other regions examined (Fig. 6d).

## Discussion

Accumulation of the PER2 protein in the cytoplasm is necessary for its effective negative regulation of molecular clock genes. Previous studies have indicated the importance of miRNAs in regulating the expression of the circadian clock gene *Per2* through posttranscriptional modification. Among the many candidate miRNAs targeting *Per2* expression, miR-24-3p and miR-30a-5p were previously reported^7,13^. In the present study, we demonstrated the modulatory function of a novel miRNA, miR-25-3p, in the regulation of *Per2* at the posttranscriptional level. Overexpression of mature miR-25-3p led to a shortened period length and dampened amplitude of PER2 oscillation, while inhibition of miR-25-3p resulted in a lengthened period with a heightened amplitude of PER2 oscillation. Based on the predictions from the TargetScan 7.2 in silico databases and the functional validation made with mutant luciferase reporter assays, miR-25-3p was found to be involved in modulating the expression level of *Per2* by targeting its seed-complimentary sequence on the 3′-UTR of *Per2* mRNA. The comparative study between miR-25-3p and miR-24-3p showed that miR-25-3p altered *Per2* circadian rhythmicity, compared to that of miR-24-3p, to a significant degree.

miRNAs are known to interact with their seed-complementary sequences on multiple target mRNAs and alter gene expression at the posttranscriptional level^23,24^. In addition, miRNAs also can induce differential effects on target mRNA expression via various binding affinities^9,10^. Our findings from the site-directed mutagenesis study showed that miR-25-3p and miR-24-3p can synergistically modulate the expression of PER2 oscillation, thereby explaining the multiplicity of miRNA function and their contributions in the fine-tuned regulation of circadian gene expression. Moreover, the results hint at the existence of various local clock oscillations in central and peripheral regions^17,25,26^, which may be generated by the posttranscriptional regulation by miRNAs. Our real-time qPCR results indicated different expression levels of miR-25-3p and miR-24-3p in various brain tissues and peripheral organs, which may have generated different modulatory effects on *Per2* expression through various combinational effects of the miRNAs targeting *Per2* expression. For instance, coherent circadian *Per2* and *Bmal1* anti-phasic gene expression was observed in both the SCN and hippocampus, showing different peak time points of *Per2* in each region (Fig. 6), and PER2 protein expression was observed in the cerebellum and liver^13^. These findings suggest plausible region-specific miR-24-3p and miR-25-3p functions in modulating local *Per2* oscillation, consequently producing individual local *Per2* oscillations in different tissue regions.

These modulatory functions of miRNAs have also been reported using DICER knockouts (KO) with loss of all the miRNAs in vitro and animal models^7,27^. Interestingly, these previous studies showed the persistent generation of *Per2* gene oscillation at the molecular level and circadian locomotor activity in the DICER-KO animals even when the total miRNAs were genetically removed. Together, these findings support the suggestion that miRNAs are involved in the fine-tuning of circadian profiles rather than in generating circadian rhythmicity^28^. In addition, the results obtained by eliminating the specific modulatory functions of miRNAs on *Per2* expression by ablating endogenous *Per2* 3′-UTR in an animal model (Per2::LucSV knock in transgenic animal) showed that there was a delayed phase shift of PER2 expression^13^. Notably, the phase delays of Per2::LucSV knock in ex vivo SCN cultures seemed to be governed by the heterozygosity of the Per2 3′-UTR, because the homozygous mutants of the Per2 3′-UTR showed a significant phase delay compared with the heterozygous mutants and wild-type. This result indicates that the posttranscriptional regulation of *Per2* expression is also dominated by copy number variations. This finding prompted us to speculate that the endogenous levels of miR-25-3p and miR-24-3p are crucial for their modulatory function and that this criterion is a possible explanation for the differential changes in the dose-dependent overexpression of miR-25-3p or miR-24-3p (Fig. 3a–d) and the great dampening of the amplitude of PER2 oscillation achieved in ex vivo SCN OE lentiviral miR-25-3p and/or miR-24-3p without altering the period lengths (Fig. 5). This outcome may be the result of a limited amount of effective miRNAs available for the posttranscriptional modification necessary to elicit changes in the *Per2* period (Fig. S2). Moreover, these results indicate that there were potential compensatory factors within neuronal networks in the SCN culture that maintained the central circadian pace-making system. It is possible that an endogenous homeostatic system regulated the concentration of mature miR-25-3p and miR-24-3p in the SCN slice culture. To understand this, more extensive studies are required to comprehend the variations in miRNA effects on gene expression in vivo.

Recently, miRNAs have attracted considerable attention in the research field because of their functional connections to cancer. Interestingly, miR-25-3p and miR-24-3p are highly involved in tumorigenesis and have been extensively studied as biomarkers for cancer progression, as reported in recent literature^29,30^. Among genes with a high score predicting targeting by miR-25-3p, B-cell translocation gene 2 (BTG2) is related to triple negative breast cancer, and miR-25-3p promotes the proliferation of breast cancer^31^. Moreover, miR-24-3p targets the gene SOX7, which is involved in the progression of lung cancer^32^. These results may link the relationship between cancer progression and disrupted circadian rhythms as regulated by similar miRNAs, such as miR-25-3p and miR-24-3p.

The present study employed gain-of-function and loss-of-function approach to understand the modulatory function of miR-25-3p on circadian *Per2* gene expression. In addition to those of other previous studies, our results showed the synergistic effects of multiple miRNAs in the fine-tuning of *Per2* rhythmicity at approximately 24 h, and this regulation might vary in different tissues. In conclusion, our study shows that miR-25-3p can modulate the expression of *Per2* through posttranscriptional modification in addition to miR-24-3p and miR-30a-5p^7,27^. These miRNAs together function to fine-tune the circadian rhythm.

## Supplementary information

Supplementary figure S1, S2, and data S3
