# Supplementary figures and images for: microRNA-25 as a novel modulator of circadian Period2 gene oscillation
Source: Exp Mol Med. 2020 Sep 23;52(9):1614–26. doi: 10.1038/s12276-020-00496-5 (PMC8080691; doi:10.1038/s12276-020-00496-5)

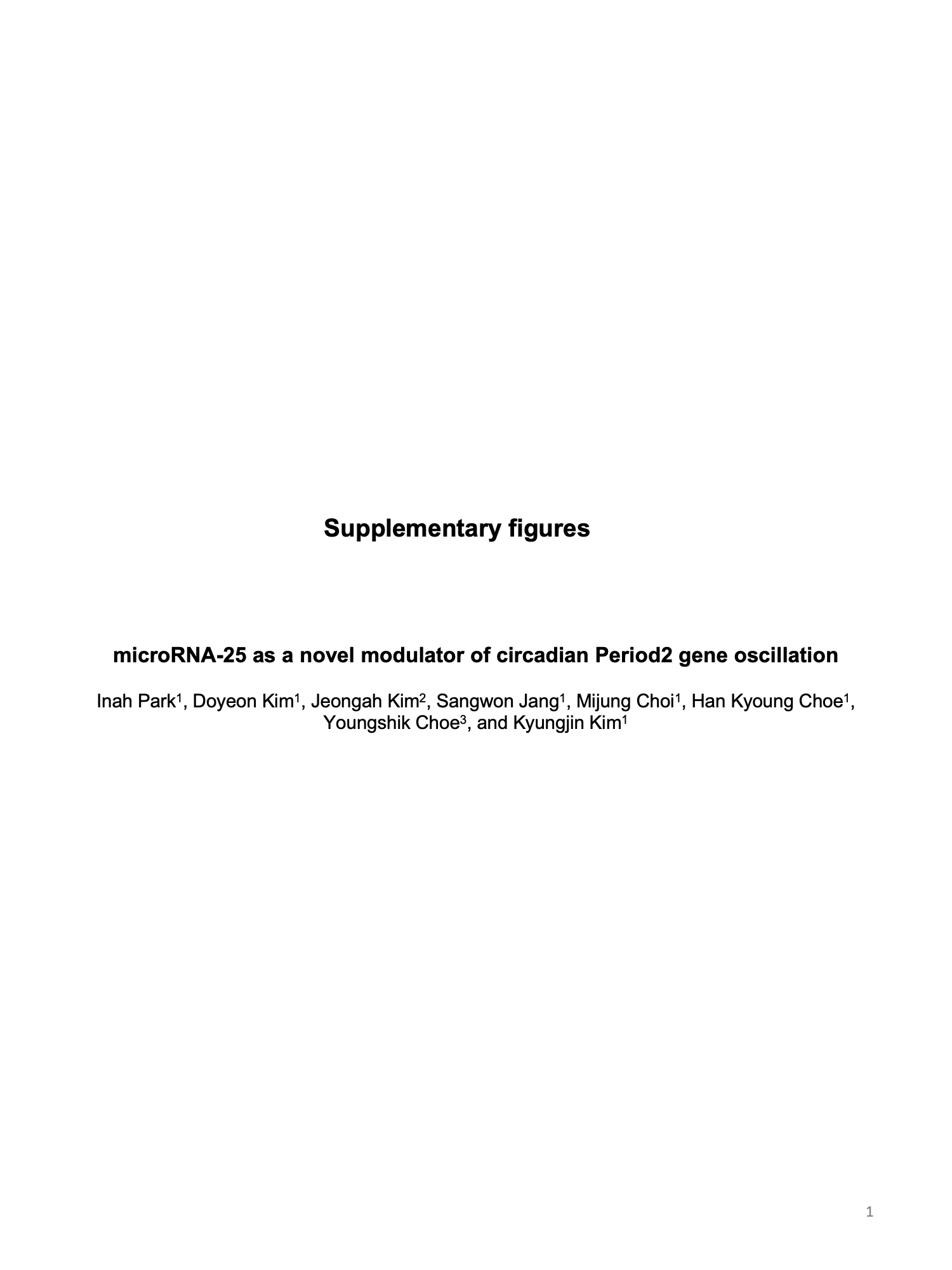

Supplement: Supplementary file 1 — Supplementary figure S1, S2, and data S3 [file 12276_2020_496_MOESM1_ESM.tif]
